# [1*H*-1,2,4-Triazole-5(4*H*)-thione-κ*S*]bis­(tri­phenyl­phosphane-κ*P*)(nitrato-κ*O*)silver(I) methanol monosolvate

**DOI:** 10.1107/S1600536814001196

**Published:** 2014-01-22

**Authors:** Yupa Wattanakanjana, Sureeporn Palamae, Jenejira Ratthiwan, Ruthairat Nimthong

**Affiliations:** a Department of Chemistry, Faculty of Science, Prince of Songkla University, Hat Yai, Songkhla 90112, Thailand

## Abstract

In the title complex, [Ag(NO_3_)(C_2_H_3_N_3_S)(C_18_H_15_P)_2_]·CH_3_OH, the Ag^I^ ion exhibits a distorted tetra­hedral coordination geometry formed by two P atoms from two tri­phenyl­phosphine ligands, one S atom from a 1*H*-1,2,4-triazole-5(4*H*)-thione ligand and one O atom from a nitrate ion. In the crystal, complex and solvent mol­ecules are linked by O—H⋯O and N—H⋯O hydrogen bonds forming a chain along the *b-*axis direction. The chains are linked by weak C—H⋯O hydrogen bonds forming a two-dimensional supra­molecular architecture parallel to (001). In addition, an intra­molecular N—H⋯O hydrogen bond is observed.

## Related literature   

For applications of 1,2,4-triazoles and their derivatives, see: Holla *et al.* (1998 ▶); Jones *et al.* (1988 ▶); Kömürcü *et al.* (1995 ▶); Küçükgüzel *et al.* (2001 ▶); Wujec & Paneth (2007 ▶). For applications of silver(I) complexes with phospho­rus and sulfur donor ligands, see: Ferrari *et al.* (2007 ▶); Isab *et al.* (2010 ▶). For related examples of discrete complexes, see: Nomiya *et al.* (1998 ▶); Pakawatchai *et al.* (2012 ▶).
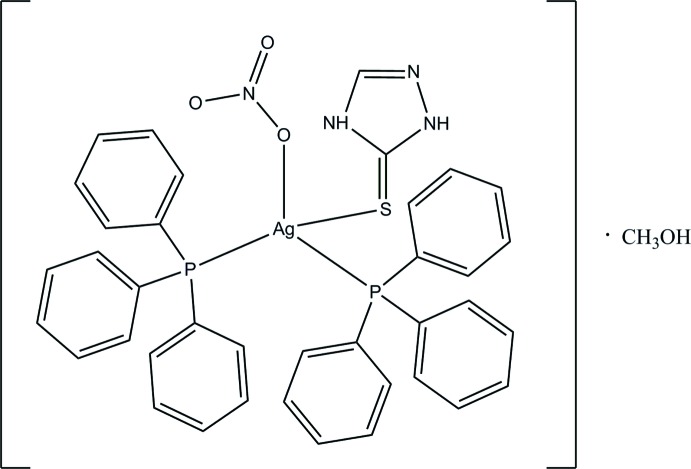


## Experimental   

### 

#### Crystal data  

[Ag(NO_3_)(C_2_H_3_N_3_S)(C_18_H_15_P)_2_]·CH_4_O
*M* *_r_* = 827.59
Monoclinic, 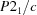

*a* = 13.2712 (14) Å
*b* = 14.3999 (15) Å
*c* = 20.198 (2) Å
β = 107.934 (2)°
*V* = 3672.4 (7) Å^3^
*Z* = 4
Mo *K*α radiation
μ = 0.74 mm^−1^
*T* = 100 K
0.37 × 0.19 × 0.18 mm

#### Data collection  

Bruker SMART APEX CCD diffractometer
Absorption correction: multi-scan (*SADABS*; Bruker, 2011 ▶) *T*_min_ = 0.644, *T*_max_ = 0.746
28287 measured reflections
10825 independent reflections
8714 reflections with *I* > 2σ(*I*)
*R*_int_ = 0.051

#### Refinement  

*R*[*F* ^2^ > 2σ(*F* ^2^)] = 0.041
*wR*(*F* ^2^) = 0.100
*S* = 1.02
10825 reflections
462 parameters
H-atom parameters constrained
Δρ_max_ = 1.35 e Å^−3^
Δρ_min_ = −0.73 e Å^−3^

### 

Data collection: *APEX2* (Bruker, 2011 ▶); cell refinement: *SAINT* (Bruker, 2011 ▶); data reduction: *SAINT*; program(s) used to solve structure: *SHELXS97* (Sheldrick, 2008 ▶); program(s) used to refine structure: *SHELXL2012* (Sheldrick, 2008 ▶) and *SHELXLE* (Hübschle *et al.*, 2011 ▶); molecular graphics: *Mercury* (Macrae *et al.*, 2008 ▶) and *PLATON* (Spek, 2009 ▶); software used to prepare material for publication: *SHELXTL* (Sheldrick, 2008 ▶).

## Supplementary Material

Crystal structure: contains datablock(s) I. DOI: 10.1107/S1600536814001196/lh5682sup1.cif

Structure factors: contains datablock(s) I. DOI: 10.1107/S1600536814001196/lh5682Isup2.hkl

CCDC reference:

Additional supporting information:  crystallographic information; 3D view; checkCIF report

## Figures and Tables

**Table 1:** Hydrogen-bond geometry (Å, °)

*D*—H⋯*A*	*D*—H	H⋯*A*	*D*⋯*A*	*D*—H⋯*A*
O4—H4⋯O1^i^	0.84	2.01	2.836 (2)	168
N1—H1⋯O2	0.88	1.93	2.793 (2)	167
N3—H3⋯O4	0.88	1.91	2.769 (3)	166
C35—H35⋯O1^i^	0.95	2.55	3.360 (3)	143
C65—H65⋯O3^ii^	0.95	2.48	3.340 (3)	150

Symmetry codes: (i) 
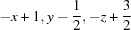
; (ii) 
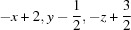
.

**Fig. 1.**
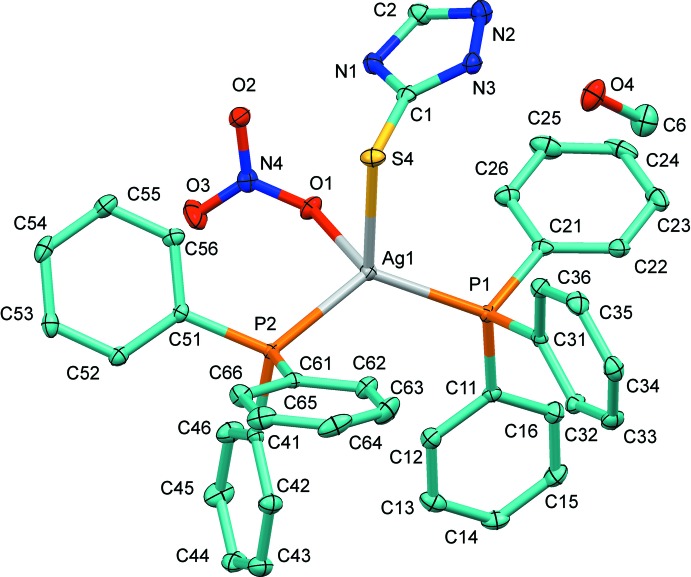
The molecular structure with displacement ellipsoids drawn at the 50% probability level. All hydrogen atoms are omitted for clarity.

**Fig. 2.**
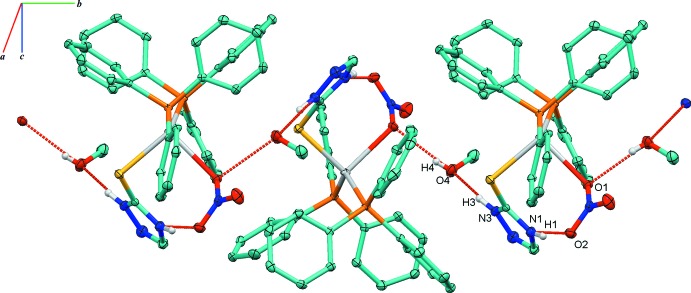
Part of the crystal structure showing intermolecular hydrogen bonds (red dashed lines) forming a 1-D chain.

**Fig. 3.**
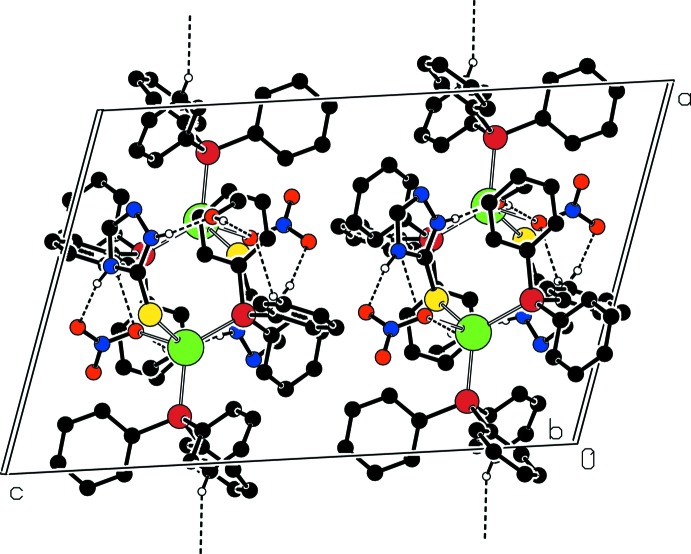
Part of the crystal structure with hydrogen bonds shown as dashed lines.

## supplementary crystallographic information

### 1. Comment

1,2,4-Triazoles and their derivatives are compounds of considerable inter­est because of variety of biological properties such as anti­microbial, anti­viral, anti­convulsant, activities, anti fungal and anti­tumor (Holla *et al.*, 1998; Jones *et al.*, 1988; Kömürcü *et al.*, 1995; Küçükgüzel *et al.*, 2001) and also potent inhibitors of enzymes. Therefore, some are approved as drugs, for example, alprazolam, etizolam, or vibrunazole (Wujec & Paneth, 2007).

The coordination chemistry of silver(I) complexes with phospho­rus and sulfur donor ligands has attracted great inter­est in recent years because of their potential applications due to anti­microbial activities and they also often show inter­esting luminescence properties (Ferrari *et al.*, 2007; Isab *et al.*, 2010). Herein, the crystal structure of a mononuclear silver(I) nitrate complex containing both tri­phenyl­phosphine and 1*H*-1,2,4-triazole-5(4*H*)-thione is described.

The molecular structure of the title compound (I) reveals the presence of tri­phenyl­phosphine and 2,4-di­hydro-3*H*-1,2,4-triazole-3-thione as co-ligands coordinated to the metal ion with two P atoms from two tri­phenyl­phosphine ligands, one terminal S atom from the 1*H*-1,2,4-triazole-5(4*H*)-thione ligand and one O atom from nitrate ion as well as one solvent methanol molecule, resulting in a distorted tetra­hedral geometry as shown in Fig. 1. The Ag—S bond distance of 2.5591 (6) Å is shorter than in two other structures [Ag(Htsa)(PPh_3_)_3_] (2.608 (7) Å, Nomiya *et al.*, 1998) and [AgBr(C_3_H_6_N_2_OS)(C_18_H_15_P)_2_] (2.8789 (10) Å, Pakawatchai *et al.*, 2012). In the crystal, hydrogen bonds play an important role with the nitrate ion connected to the methanol molecule with inter­molecular O4—H4···O1^i^, N1—H1···O2 and N3—H3···O4 inter­actions (see Table 1) leading to the formation of a 1-D chain along [010], Fig. 2. Furthermore, chains are linked by weak C—H···O hydrogen bonds forming of a 2-D supra­molecular architecture parallel to (001). In addition, an intra­molecular N—H···O hydrogen bond is observed (Fig. 3).

### 2. Experimental

Tri­phenyl­phosphine, PPh_3_, (0.31g,1.18 mmol) was dissolved in 30 cm^3^ of methanol at 333 K. AgNO_3_ (0.10g,0.59 mmol) was added and the mixture was stirred for 3 hours. 1*H*-1,2,4-Triazole-5(4*H*)-thione, (0.06g,0.59 mmol) was added and new reaction mixture was heated under reflux for 3 hours. The resulting clear solution was filtered off and left to evaporate at room temperature. Colorless crystal, which was deposited upon standing for few days, was filtered off and dried under reduced pressure.

### 3. Refinement

H atoms bonded to C, N and O atoms were constrained with a riding model of of 0.95 Å (aryl H), and *U*_iso_(H) = 1.2*U*_eq_(C); 0.98 Å(CH_3_) and *U*_iso_(H) = 1.5*U*_eq_(C); 0.88 Å(NH) and *U*_iso_(H) = 1.2*U*_eq_(N); 0.84 Å(OH) and *U*_iso_(H) = 1.5*U*_eq_(O). Reflections 0 1 1, 1 0 0, 16 4 4, -7 8 2, -2 9 25, 7 1 0 were affected by the beam stop and were omitted from the refinement.

### Crystal data

**Table:**

[Ag(NO_3_)(C_2_H_3_N_3_S)(C_18_H_15_P)_2_]·CH_4_O	*F*(000) = 1696
*M**_r_* = 827.59	*D*_x_ = 1.497 Mg m^−^^3^
Monoclinic, *P*2_1_/*c*	Mo *K*α radiation, λ = 0.71073 Å
*a* = 13.2712 (14) Å	Cell parameters from 6153 reflections
*b* = 14.3999 (15) Å	θ = 2.2–30.1°
*c* = 20.198 (2) Å	µ = 0.74 mm^−^^1^
β = 107.934 (2)°	*T* = 100 K
*V* = 3672.4 (7) Å^3^	Block, colourless
*Z* = 4	0.37 × 0.19 × 0.18 mm

### Data collection

**Table:**

Bruker SMART APEX CCD diffractometer	8714 reflections with *I* > 2σ(*I*)
Radiation source: fine focus sealed tube	*R*_int_ = 0.051
ω and phi scans	θ_max_ = 31.4°, θ_min_ = 2.2°
Absorption correction: multi-scan (*SADABS*; Bruker, 2011)	*h* = −19→18
*T*_min_ = 0.644, *T*_max_ = 0.746	*k* = −17→20
28287 measured reflections	*l* = −28→25
10825 independent reflections	

### Refinement

**Table:**

Refinement on *F*^2^	Primary atom site location: structure-invariant direct methods
Least-squares matrix: full	Secondary atom site location: difference Fourier map
*R*[*F*^2^ > 2σ(*F*^2^)] = 0.041	Hydrogen site location: inferred from neighbouring sites
*wR*(*F*^2^) = 0.100	H-atom parameters constrained
*S* = 1.02	*w* = 1/[σ^2^(*F*_o_^2^) + (0.0459*P*)^2^ + 0.1882*P*] where *P* = (*F*_o_^2^ + 2*F*_c_^2^)/3
10825 reflections	(Δ/σ)_max_ = 0.003
462 parameters	Δρ_max_ = 1.35 e Å^−^^3^
0 restraints	Δρ_min_ = −0.73 e Å^−^^3^

### Special details

**Table:**

Geometry. All e.s.d.'s (except the e.s.d. in the dihedral angle between two l.s. planes) are estimated using the full covariance matrix. The cell e.s.d.'s are taken into account individually in the estimation of e.s.d.'s in distances, angles and torsion angles; correlations between e.s.d.'s in cell parameters are only used when they are defined by crystal symmetry. An approximate (isotropic) treatment of cell e.s.d.'s is used for estimating e.s.d.'s involving l.s. planes.

### Fractional atomic coordinates and isotropic or equivalent isotropic displacement parameters (Å^2^)

**Table:**

	*x*	*y*	*z*	*U*_iso_*/*U*_eq_	
Ag1	0.67429 (2)	0.93151 (2)	0.76336 (2)	0.01160 (5)	
S1	0.57664 (4)	0.80068 (4)	0.68338 (3)	0.01644 (12)	
P1	0.59184 (4)	0.97770 (4)	0.85134 (3)	0.01086 (11)	
P2	0.86540 (4)	0.91525 (4)	0.78475 (3)	0.01009 (11)	
C2	0.32936 (18)	0.93273 (16)	0.57203 (13)	0.0181 (5)	
H2	0.2941	0.9761	0.5371	0.022*	
N2	0.28180 (15)	0.88131 (14)	0.60609 (11)	0.0197 (4)	
O1	0.62092 (13)	1.05965 (11)	0.66828 (8)	0.0174 (3)	
O2	0.60167 (13)	1.01016 (12)	0.56286 (8)	0.0194 (4)	
O3	0.72933 (14)	1.10506 (14)	0.61295 (10)	0.0299 (4)	
O4	0.31291 (15)	0.71893 (13)	0.74684 (9)	0.0258 (4)	
H4	0.3255	0.6677	0.7677	0.039*	
N1	0.43582 (15)	0.91708 (13)	0.59233 (10)	0.0155 (4)	
H1	0.4822	0.9453	0.5761	0.019*	
N3	0.36316 (14)	0.82974 (14)	0.64945 (10)	0.0160 (4)	
H3	0.3541	0.7879	0.6788	0.019*	
N4	0.65185 (15)	1.05927 (13)	0.61445 (11)	0.0158 (4)	
C1	0.45739 (17)	0.85036 (16)	0.64198 (11)	0.0138 (4)	
C6	0.2732 (2)	0.78183 (19)	0.78606 (14)	0.0275 (6)	
H6A	0.2589	0.8417	0.7619	0.041*	
H6B	0.3256	0.7904	0.8319	0.041*	
H6C	0.2075	0.7572	0.7917	0.041*	
C11	0.65211 (16)	1.07206 (15)	0.91082 (11)	0.0115 (4)	
C12	0.75475 (17)	1.09950 (16)	0.91578 (12)	0.0149 (4)	
H12	0.7907	1.0710	0.8871	0.018*	
C13	0.80489 (18)	1.16858 (16)	0.96255 (13)	0.0188 (5)	
H13	0.8753	1.1865	0.9661	0.023*	
C14	0.75264 (19)	1.21114 (16)	1.00377 (12)	0.0189 (5)	
H14	0.7874	1.2576	1.0361	0.023*	
C15	0.64965 (19)	1.18617 (17)	0.99799 (12)	0.0203 (5)	
H15	0.6133	1.2164	1.0258	0.024*	
C16	0.59928 (18)	1.11716 (17)	0.95173 (12)	0.0178 (5)	
H16	0.5284	1.1004	0.9478	0.021*	
C21	0.45256 (16)	1.00891 (15)	0.81942 (12)	0.0132 (4)	
C22	0.38246 (17)	0.99700 (17)	0.85794 (12)	0.0180 (5)	
H22	0.4051	0.9664	0.9017	0.022*	
C23	0.27920 (18)	1.03002 (19)	0.83202 (14)	0.0239 (6)	
H23	0.2317	1.0223	0.8584	0.029*	
C24	0.24512 (19)	1.07409 (17)	0.76800 (15)	0.0256 (6)	
H24	0.1750	1.0977	0.7510	0.031*	
C25	0.3138 (2)	1.08353 (18)	0.72893 (15)	0.0262 (6)	
H25	0.2902	1.1123	0.6845	0.031*	
C26	0.41716 (19)	1.05108 (16)	0.75449 (13)	0.0187 (5)	
H26	0.4639	1.0578	0.7274	0.022*	
C31	0.59714 (15)	0.87695 (15)	0.90766 (11)	0.0112 (4)	
C32	0.63638 (17)	0.87995 (16)	0.97996 (12)	0.0150 (4)	
H32	0.6606	0.9373	1.0025	0.018*	
C33	0.64067 (18)	0.80025 (17)	1.01962 (12)	0.0177 (5)	
H33	0.6672	0.8032	1.0689	0.021*	
C34	0.60578 (17)	0.71596 (17)	0.98657 (13)	0.0176 (5)	
H34	0.6081	0.6613	1.0134	0.021*	
C35	0.56759 (18)	0.71196 (17)	0.91447 (13)	0.0189 (5)	
H35	0.5440	0.6545	0.8920	0.023*	
C36	0.56372 (17)	0.79166 (16)	0.87521 (12)	0.0161 (5)	
H36	0.5382	0.7883	0.8259	0.019*	
C41	0.95262 (15)	1.00163 (15)	0.83888 (11)	0.0115 (4)	
C42	1.02105 (17)	0.98262 (17)	0.90534 (12)	0.0166 (5)	
H42	1.0261	0.9213	0.9234	0.020*	
C43	1.08189 (19)	1.05348 (17)	0.94521 (13)	0.0193 (5)	
H43	1.1275	1.0403	0.9906	0.023*	
C44	1.07645 (18)	1.14226 (17)	0.91939 (12)	0.0191 (5)	
H44	1.1188	1.1901	0.9467	0.023*	
C45	1.0089 (2)	1.16192 (17)	0.85322 (13)	0.0230 (5)	
H45	1.0051	1.2231	0.8351	0.028*	
C46	0.94721 (19)	1.09224 (17)	0.81390 (13)	0.0194 (5)	
H46	0.9003	1.1063	0.7690	0.023*	
C51	0.91175 (16)	0.90988 (15)	0.70926 (11)	0.0115 (4)	
C52	1.01807 (18)	0.92341 (15)	0.71397 (12)	0.0141 (4)	
H52	1.0682	0.9377	0.7577	0.017*	
C53	1.05087 (18)	0.91613 (16)	0.65508 (13)	0.0169 (5)	
H53	1.1234	0.9242	0.6586	0.020*	
C54	0.97678 (19)	0.89701 (17)	0.59094 (12)	0.0188 (5)	
H54	0.9989	0.8914	0.5506	0.023*	
C55	0.87081 (19)	0.88607 (17)	0.58562 (12)	0.0187 (5)	
H55	0.8203	0.8751	0.5415	0.022*	
C56	0.83820 (17)	0.89111 (16)	0.64467 (11)	0.0143 (4)	
H56	0.7658	0.8818	0.6410	0.017*	
C61	0.90777 (16)	0.80667 (15)	0.83114 (11)	0.0120 (4)	
C62	0.85267 (18)	0.77704 (16)	0.87603 (12)	0.0170 (5)	
H62	0.7950	0.8126	0.8807	0.020*	
C63	0.8822 (2)	0.69565 (18)	0.91380 (13)	0.0247 (5)	
H63	0.8450	0.6759	0.9447	0.030*	
C64	0.9654 (2)	0.64320 (17)	0.90676 (12)	0.0238 (5)	
H64	0.9850	0.5875	0.9327	0.029*	
C65	1.02054 (19)	0.67137 (17)	0.86202 (13)	0.0208 (5)	
H65	1.0775	0.6350	0.8571	0.025*	
C66	0.99194 (17)	0.75288 (16)	0.82467 (12)	0.0171 (5)	
H66	1.0299	0.7725	0.7943	0.020*	

### Atomic displacement parameters (Å^2^)

**Table:**

	*U*^11^	*U*^22^	*U*^33^	*U*^12^	*U*^13^	*U*^23^
Ag1	0.01102 (8)	0.01409 (9)	0.00990 (9)	0.00083 (6)	0.00351 (6)	−0.00047 (6)
S1	0.0177 (2)	0.0141 (3)	0.0152 (3)	0.0004 (2)	0.0017 (2)	−0.0025 (2)
P1	0.0107 (2)	0.0125 (3)	0.0096 (3)	0.00039 (19)	0.0035 (2)	−0.0006 (2)
P2	0.0097 (2)	0.0115 (3)	0.0091 (3)	0.00054 (19)	0.0030 (2)	0.0003 (2)
C2	0.0169 (10)	0.0174 (12)	0.0193 (12)	0.0005 (8)	0.0046 (9)	0.0014 (9)
N2	0.0178 (9)	0.0207 (11)	0.0218 (11)	0.0012 (8)	0.0080 (8)	0.0008 (9)
O1	0.0242 (8)	0.0153 (9)	0.0136 (8)	0.0014 (6)	0.0072 (7)	0.0024 (7)
O2	0.0215 (8)	0.0229 (9)	0.0142 (8)	−0.0053 (7)	0.0058 (7)	−0.0018 (7)
O3	0.0267 (9)	0.0294 (11)	0.0384 (12)	−0.0152 (8)	0.0170 (9)	−0.0066 (9)
O4	0.0398 (11)	0.0192 (10)	0.0229 (10)	0.0015 (8)	0.0162 (9)	0.0046 (7)
N1	0.0151 (8)	0.0159 (10)	0.0160 (10)	−0.0016 (7)	0.0054 (8)	0.0017 (8)
N3	0.0175 (9)	0.0169 (10)	0.0144 (10)	−0.0032 (7)	0.0060 (7)	0.0014 (8)
N4	0.0177 (9)	0.0122 (10)	0.0186 (10)	0.0008 (7)	0.0071 (8)	0.0025 (8)
C1	0.0175 (10)	0.0122 (11)	0.0116 (10)	−0.0033 (8)	0.0044 (8)	−0.0040 (9)
C6	0.0324 (14)	0.0230 (14)	0.0301 (15)	0.0051 (11)	0.0139 (12)	0.0035 (12)
C11	0.0120 (9)	0.0117 (10)	0.0098 (10)	0.0008 (7)	0.0019 (8)	0.0005 (8)
C12	0.0157 (10)	0.0142 (11)	0.0148 (11)	0.0018 (8)	0.0048 (9)	0.0003 (9)
C13	0.0156 (10)	0.0147 (12)	0.0228 (13)	0.0001 (8)	0.0011 (9)	0.0017 (10)
C14	0.0231 (11)	0.0138 (12)	0.0157 (12)	−0.0009 (9)	0.0002 (9)	−0.0026 (9)
C15	0.0260 (12)	0.0201 (13)	0.0158 (12)	0.0023 (9)	0.0079 (10)	−0.0043 (10)
C16	0.0163 (10)	0.0203 (12)	0.0179 (12)	−0.0005 (9)	0.0066 (9)	−0.0034 (10)
C21	0.0125 (9)	0.0123 (11)	0.0131 (11)	0.0015 (8)	0.0012 (8)	−0.0030 (9)
C22	0.0141 (10)	0.0237 (13)	0.0149 (12)	−0.0017 (9)	0.0023 (9)	−0.0054 (10)
C23	0.0143 (10)	0.0299 (15)	0.0290 (15)	−0.0020 (10)	0.0089 (10)	−0.0138 (12)
C24	0.0125 (10)	0.0202 (13)	0.0364 (16)	0.0028 (9)	−0.0037 (10)	−0.0102 (11)
C25	0.0205 (12)	0.0220 (14)	0.0296 (16)	0.0034 (10)	−0.0018 (11)	0.0034 (11)
C26	0.0199 (11)	0.0175 (12)	0.0169 (12)	0.0000 (9)	0.0032 (9)	0.0008 (10)
C31	0.0091 (8)	0.0125 (10)	0.0123 (11)	0.0018 (7)	0.0038 (8)	0.0011 (8)
C32	0.0161 (10)	0.0155 (11)	0.0132 (11)	−0.0002 (8)	0.0044 (8)	−0.0025 (9)
C33	0.0193 (10)	0.0207 (12)	0.0119 (11)	0.0018 (9)	0.0030 (9)	0.0034 (9)
C34	0.0158 (10)	0.0164 (12)	0.0217 (13)	0.0012 (8)	0.0072 (9)	0.0050 (10)
C35	0.0197 (11)	0.0154 (12)	0.0227 (13)	−0.0043 (9)	0.0083 (10)	−0.0036 (10)
C36	0.0168 (10)	0.0176 (12)	0.0142 (11)	−0.0029 (8)	0.0053 (9)	−0.0010 (9)
C41	0.0103 (9)	0.0131 (11)	0.0116 (10)	0.0008 (8)	0.0039 (8)	−0.0001 (9)
C42	0.0175 (10)	0.0152 (12)	0.0154 (12)	0.0001 (8)	0.0024 (9)	0.0019 (9)
C43	0.0197 (11)	0.0196 (13)	0.0157 (12)	−0.0018 (9)	0.0014 (9)	0.0003 (10)
C44	0.0220 (11)	0.0168 (12)	0.0175 (12)	−0.0040 (9)	0.0047 (10)	−0.0055 (10)
C45	0.0352 (13)	0.0113 (12)	0.0206 (13)	−0.0032 (10)	0.0058 (11)	0.0009 (10)
C46	0.0265 (12)	0.0140 (12)	0.0151 (12)	0.0000 (9)	0.0024 (10)	0.0012 (9)
C51	0.0140 (9)	0.0104 (10)	0.0110 (10)	0.0007 (8)	0.0052 (8)	0.0009 (8)
C52	0.0169 (10)	0.0140 (11)	0.0118 (11)	−0.0005 (8)	0.0051 (9)	0.0008 (9)
C53	0.0190 (10)	0.0140 (11)	0.0215 (13)	−0.0017 (8)	0.0117 (9)	0.0018 (9)
C54	0.0290 (12)	0.0163 (12)	0.0156 (12)	0.0000 (9)	0.0136 (10)	−0.0008 (10)
C55	0.0245 (11)	0.0188 (12)	0.0126 (11)	0.0037 (9)	0.0057 (9)	0.0005 (9)
C56	0.0153 (10)	0.0140 (11)	0.0122 (11)	0.0017 (8)	0.0021 (8)	−0.0001 (9)
C61	0.0132 (9)	0.0109 (10)	0.0100 (10)	−0.0017 (8)	0.0010 (8)	−0.0014 (8)
C62	0.0214 (11)	0.0172 (12)	0.0129 (11)	0.0005 (9)	0.0059 (9)	0.0004 (9)
C63	0.0387 (14)	0.0223 (14)	0.0153 (12)	−0.0009 (11)	0.0117 (11)	0.0042 (10)
C64	0.0418 (15)	0.0114 (12)	0.0127 (12)	0.0043 (10)	0.0005 (11)	0.0020 (9)
C65	0.0244 (12)	0.0153 (12)	0.0194 (13)	0.0060 (9)	0.0019 (10)	−0.0025 (10)
C66	0.0164 (10)	0.0150 (12)	0.0196 (12)	0.0016 (8)	0.0052 (9)	0.0010 (9)

### Geometric parameters (Å, º)

**Table:**

Ag1—P1	2.4485 (6)	C25—H25	0.9500
Ag1—P2	2.4493 (6)	C26—H26	0.9500
Ag1—S1	2.5591 (6)	C31—C32	1.392 (3)
Ag1—O1	2.5994 (16)	C31—C36	1.398 (3)
S1—C1	1.703 (2)	C32—C33	1.391 (3)
P1—C21	1.817 (2)	C32—H32	0.9500
P1—C11	1.827 (2)	C33—C34	1.394 (3)
P1—C31	1.832 (2)	C33—H33	0.9500
P2—C51	1.813 (2)	C34—C35	1.388 (3)
P2—C41	1.817 (2)	C34—H34	0.9500
P2—C61	1.821 (2)	C35—C36	1.387 (3)
C2—N2	1.299 (3)	C35—H35	0.9500
C2—N1	1.363 (3)	C36—H36	0.9500
C2—H2	0.9500	C41—C46	1.393 (3)
N2—N3	1.379 (3)	C41—C42	1.397 (3)
O1—N4	1.275 (2)	C42—C43	1.393 (3)
O2—N4	1.266 (3)	C42—H42	0.9500
O3—N4	1.230 (2)	C43—C44	1.374 (3)
O4—C6	1.408 (3)	C43—H43	0.9500
O4—H4	0.8400	C44—C45	1.390 (3)
N1—C1	1.354 (3)	C44—H44	0.9500
N1—H1	0.8800	C45—C46	1.381 (3)
N3—C1	1.338 (3)	C45—H45	0.9500
N3—H3	0.8800	C46—H46	0.9500
C6—H6A	0.9800	C51—C56	1.394 (3)
C6—H6B	0.9800	C51—C52	1.398 (3)
C6—H6C	0.9800	C52—C53	1.391 (3)
C11—C12	1.392 (3)	C52—H52	0.9500
C11—C16	1.397 (3)	C53—C54	1.391 (3)
C12—C13	1.392 (3)	C53—H53	0.9500
C12—H12	0.9500	C54—C55	1.386 (3)
C13—C14	1.380 (3)	C54—H54	0.9500
C13—H13	0.9500	C55—C56	1.390 (3)
C14—C15	1.383 (3)	C55—H55	0.9500
C14—H14	0.9500	C56—H56	0.9500
C15—C16	1.387 (3)	C61—C62	1.395 (3)
C15—H15	0.9500	C61—C66	1.398 (3)
C16—H16	0.9500	C62—C63	1.388 (3)
C21—C26	1.389 (3)	C62—H62	0.9500
C21—C22	1.395 (3)	C63—C64	1.381 (4)
C22—C23	1.392 (3)	C63—H63	0.9500
C22—H22	0.9500	C64—C65	1.387 (4)
C23—C24	1.385 (4)	C64—H64	0.9500
C23—H23	0.9500	C65—C66	1.384 (3)
C24—C25	1.384 (4)	C65—H65	0.9500
C24—H24	0.9500	C66—H66	0.9500
C25—C26	1.390 (3)		
			
P1—Ag1—P2	124.60 (2)	C21—C26—C25	120.4 (2)
P1—Ag1—S1	113.92 (2)	C21—C26—H26	119.8
P2—Ag1—S1	109.667 (19)	C25—C26—H26	119.8
P1—Ag1—O1	105.21 (4)	C32—C31—C36	118.8 (2)
P2—Ag1—O1	103.29 (4)	C32—C31—P1	123.88 (17)
S1—Ag1—O1	94.96 (4)	C36—C31—P1	117.30 (17)
C1—S1—Ag1	102.36 (8)	C33—C32—C31	121.0 (2)
C21—P1—C11	103.94 (10)	C33—C32—H32	119.5
C21—P1—C31	104.87 (10)	C31—C32—H32	119.5
C11—P1—C31	104.80 (10)	C32—C33—C34	119.6 (2)
C21—P1—Ag1	116.29 (7)	C32—C33—H33	120.2
C11—P1—Ag1	118.45 (7)	C34—C33—H33	120.2
C31—P1—Ag1	107.18 (7)	C35—C34—C33	119.9 (2)
C51—P2—C41	102.53 (10)	C35—C34—H34	120.0
C51—P2—C61	105.38 (10)	C33—C34—H34	120.0
C41—P2—C61	103.86 (10)	C36—C35—C34	120.2 (2)
C51—P2—Ag1	117.18 (7)	C36—C35—H35	119.9
C41—P2—Ag1	118.28 (7)	C34—C35—H35	119.9
C61—P2—Ag1	108.17 (7)	C35—C36—C31	120.5 (2)
N2—C2—N1	112.0 (2)	C35—C36—H36	119.7
N2—C2—H2	124.0	C31—C36—H36	119.7
N1—C2—H2	124.0	C46—C41—C42	118.5 (2)
C2—N2—N3	103.30 (18)	C46—C41—P2	118.09 (17)
N4—O1—Ag1	122.75 (13)	C42—C41—P2	123.32 (17)
C6—O4—H4	109.5	C43—C42—C41	120.1 (2)
C1—N1—C2	107.65 (19)	C43—C42—H42	119.9
C1—N1—H1	126.2	C41—C42—H42	119.9
C2—N1—H1	126.2	C44—C43—C42	120.6 (2)
C1—N3—N2	112.65 (19)	C44—C43—H43	119.7
C1—N3—H3	123.7	C42—C43—H43	119.7
N2—N3—H3	123.7	C43—C44—C45	119.8 (2)
O3—N4—O2	120.7 (2)	C43—C44—H44	120.1
O3—N4—O1	120.7 (2)	C45—C44—H44	120.1
O2—N4—O1	118.59 (18)	C46—C45—C44	119.8 (2)
N3—C1—N1	104.42 (19)	C46—C45—H45	120.1
N3—C1—S1	127.64 (18)	C44—C45—H45	120.1
N1—C1—S1	127.89 (17)	C45—C46—C41	121.1 (2)
O4—C6—H6A	109.5	C45—C46—H46	119.4
O4—C6—H6B	109.5	C41—C46—H46	119.4
H6A—C6—H6B	109.5	C56—C51—C52	119.5 (2)
O4—C6—H6C	109.5	C56—C51—P2	118.31 (16)
H6A—C6—H6C	109.5	C52—C51—P2	122.24 (17)
H6B—C6—H6C	109.5	C53—C52—C51	120.4 (2)
C12—C11—C16	118.9 (2)	C53—C52—H52	119.8
C12—C11—P1	118.62 (16)	C51—C52—H52	119.8
C16—C11—P1	122.46 (16)	C52—C53—C54	119.6 (2)
C11—C12—C13	120.3 (2)	C52—C53—H53	120.2
C11—C12—H12	119.9	C54—C53—H53	120.2
C13—C12—H12	119.9	C55—C54—C53	120.3 (2)
C14—C13—C12	120.2 (2)	C55—C54—H54	119.9
C14—C13—H13	119.9	C53—C54—H54	119.9
C12—C13—H13	119.9	C54—C55—C56	120.3 (2)
C13—C14—C15	120.0 (2)	C54—C55—H55	119.9
C13—C14—H14	120.0	C56—C55—H55	119.9
C15—C14—H14	120.0	C55—C56—C51	120.0 (2)
C14—C15—C16	120.1 (2)	C55—C56—H56	120.0
C14—C15—H15	119.9	C51—C56—H56	120.0
C16—C15—H15	119.9	C62—C61—C66	119.0 (2)
C15—C16—C11	120.4 (2)	C62—C61—P2	117.16 (17)
C15—C16—H16	119.8	C66—C61—P2	123.83 (17)
C11—C16—H16	119.8	C63—C62—C61	120.0 (2)
C26—C21—C22	119.4 (2)	C63—C62—H62	120.0
C26—C21—P1	117.04 (17)	C61—C62—H62	120.0
C22—C21—P1	123.51 (18)	C64—C63—C62	120.3 (2)
C23—C22—C21	119.8 (2)	C64—C63—H63	119.8
C23—C22—H22	120.1	C62—C63—H63	119.8
C21—C22—H22	120.1	C63—C64—C65	120.4 (2)
C24—C23—C22	120.5 (2)	C63—C64—H64	119.8
C24—C23—H23	119.7	C65—C64—H64	119.8
C22—C23—H23	119.7	C66—C65—C64	119.5 (2)
C25—C24—C23	119.6 (2)	C66—C65—H65	120.3
C25—C24—H24	120.2	C64—C65—H65	120.3
C23—C24—H24	120.2	C65—C66—C61	120.8 (2)
C24—C25—C26	120.2 (3)	C65—C66—H66	119.6
C24—C25—H25	119.9	C61—C66—H66	119.6
C26—C25—H25	119.9		
			
N1—C2—N2—N3	−0.8 (3)	C32—C33—C34—C35	0.3 (3)
N2—C2—N1—C1	0.9 (3)	C33—C34—C35—C36	−0.2 (3)
C2—N2—N3—C1	0.5 (3)	C34—C35—C36—C31	−0.6 (3)
Ag1—O1—N4—O3	−96.6 (2)	C32—C31—C36—C35	1.3 (3)
Ag1—O1—N4—O2	82.8 (2)	P1—C31—C36—C35	178.64 (17)
N2—N3—C1—N1	0.0 (3)	C51—P2—C41—C46	64.26 (19)
N2—N3—C1—S1	−177.87 (17)	C61—P2—C41—C46	173.81 (17)
C2—N1—C1—N3	−0.5 (2)	Ag1—P2—C41—C46	−66.36 (19)
C2—N1—C1—S1	177.36 (18)	C51—P2—C41—C42	−118.73 (19)
Ag1—S1—C1—N3	−110.5 (2)	C61—P2—C41—C42	−9.2 (2)
Ag1—S1—C1—N1	72.2 (2)	Ag1—P2—C41—C42	110.66 (17)
C21—P1—C11—C12	−145.97 (18)	C46—C41—C42—C43	0.1 (3)
C31—P1—C11—C12	104.21 (18)	P2—C41—C42—C43	−176.86 (18)
Ag1—P1—C11—C12	−15.2 (2)	C41—C42—C43—C44	−0.9 (4)
C21—P1—C11—C16	34.4 (2)	C42—C43—C44—C45	0.7 (4)
C31—P1—C11—C16	−75.4 (2)	C43—C44—C45—C46	0.3 (4)
Ag1—P1—C11—C16	165.24 (17)	C44—C45—C46—C41	−1.1 (4)
C16—C11—C12—C13	2.2 (3)	C42—C41—C46—C45	0.8 (3)
P1—C11—C12—C13	−177.41 (18)	P2—C41—C46—C45	178.00 (19)
C11—C12—C13—C14	−0.8 (4)	C41—P2—C51—C56	−147.58 (18)
C12—C13—C14—C15	−0.9 (4)	C61—P2—C51—C56	104.02 (18)
C13—C14—C15—C16	1.2 (4)	Ag1—P2—C51—C56	−16.3 (2)
C14—C15—C16—C11	0.3 (4)	C41—P2—C51—C52	32.8 (2)
C12—C11—C16—C15	−1.9 (3)	C61—P2—C51—C52	−75.6 (2)
P1—C11—C16—C15	177.65 (18)	Ag1—P2—C51—C52	164.03 (15)
C11—P1—C21—C26	99.22 (19)	C56—C51—C52—C53	−1.5 (3)
C31—P1—C21—C26	−151.02 (18)	P2—C51—C52—C53	178.19 (17)
Ag1—P1—C21—C26	−32.9 (2)	C51—C52—C53—C54	1.2 (3)
C11—P1—C21—C22	−77.4 (2)	C52—C53—C54—C55	0.6 (4)
C31—P1—C21—C22	32.3 (2)	C53—C54—C55—C56	−2.1 (4)
Ag1—P1—C21—C22	150.51 (17)	C54—C55—C56—C51	1.8 (4)
C26—C21—C22—C23	−2.1 (4)	C52—C51—C56—C55	−0.1 (3)
P1—C21—C22—C23	174.51 (18)	P2—C51—C56—C55	−179.74 (18)
C21—C22—C23—C24	0.5 (4)	C51—P2—C61—C62	−157.71 (17)
C22—C23—C24—C25	1.4 (4)	C41—P2—C61—C62	94.86 (18)
C23—C24—C25—C26	−1.6 (4)	Ag1—P2—C61—C62	−31.62 (19)
C22—C21—C26—C25	1.8 (4)	C51—P2—C61—C66	22.9 (2)
P1—C21—C26—C25	−174.95 (19)	C41—P2—C61—C66	−84.5 (2)
C24—C25—C26—C21	0.0 (4)	Ag1—P2—C61—C66	149.00 (17)
C21—P1—C31—C32	−106.86 (18)	C66—C61—C62—C63	0.5 (3)
C11—P1—C31—C32	2.3 (2)	P2—C61—C62—C63	−178.93 (19)
Ag1—P1—C31—C32	128.97 (16)	C61—C62—C63—C64	−0.6 (4)
C21—P1—C31—C36	75.99 (18)	C62—C63—C64—C65	0.2 (4)
C11—P1—C31—C36	−174.86 (16)	C63—C64—C65—C66	0.4 (4)
Ag1—P1—C31—C36	−48.18 (17)	C64—C65—C66—C61	−0.5 (4)
C36—C31—C32—C33	−1.2 (3)	C62—C61—C66—C65	0.0 (3)
P1—C31—C32—C33	−178.32 (17)	P2—C61—C66—C65	179.41 (18)
C31—C32—C33—C34	0.4 (3)		

### Hydrogen-bond geometry (Å, º)

**Table:**

*D*—H···*A*	*D*—H	H···*A*	*D*···*A*	*D*—H···*A*
O4—H4···O1^i^	0.84	2.01	2.836 (2)	168
N1—H1···O2	0.88	1.93	2.793 (2)	167
N3—H3···O4	0.88	1.91	2.769 (3)	166
C35—H35···O1^i^	0.95	2.55	3.360 (3)	143
C65—H65···O3^ii^	0.95	2.48	3.340 (3)	150

Symmetry codes: (i) −*x*+1, *y*−1/2, −*z*+3/2; (ii) −*x*+2, *y*−1/2, −*z*+3/2.
